# A Comparative *In Vitro* and *In Vivo* Study of Osteogenicity by Using Two Biomaterials and Two Human Mesenchymal Stem Cell Subtypes

**DOI:** 10.3389/fcell.2022.913539

**Published:** 2022-05-30

**Authors:** L. Fievet, N. Serratrice, B. Brulin, L. Giraudo, J. Véran, N. Degardin, F. Sabatier, F. Féron, P. Layrolle

**Affiliations:** ^1^ Department of Pediatric Surgery, Centre Hospitalier Régional Henri Duffaut, Avignon, France; ^2^ Department of Neurosurgery, La Timone Hospital, Assistance Publique-Hôpitaux de Marseille, Marseille, France; ^3^ APHM, Culture and Cell Therapy Laboratory, Inserm CBT-1409, Centre d’Investigations Cliniques en Biothérapies, Marseille, France; ^4^ INSERM, UMR 1238, PHY-OS, Bone Sarcomas and Remodeling of Calcified Tissues, Faculty of Medicine, Nantes University Nantes, Nantes, France; ^5^ Department of Pediatric Surgery, La Timone Enfant Hospital, Assistance Publique—Hôpitaux de Marseille, Marseille, France; ^6^ Aix Marseille University, CNRS, INP, Marseille, France

**Keywords:** bone marrow, nose, human mesenchymal stem/stromal cells, osteoinduction, biomaterials, biphasic calcium phosphate, bioactive glasses

## Abstract

**Background:** Bone repair induced by stem cells and biomaterials may represent an alternative to autologous bone grafting. Mesenchymal stromal/stem cells (MSCs), easily accessible in every human, are prototypical cells that can be tested, alone or with a biomaterial, for creating new osteoblasts. The aim of this study was to compare the efficiency of two biomaterials—biphasic calcium phosphate (BCP) and bioactive glass (BG)—when loaded with either adult bone marrow mesenchymal stem cells (BMMSCs) or newborn nasal ecto-mesenchymal stem cells (NE-MSCs), the latter being collected for further repair of lip cleft-associated bone loss.

**Materials and Methods:** BMMSCs were collected from two adults and NE-MSCs from two newborn infants. An *in vitro* study was performed in order to determine the best experimental conditions for adhesion, viability, proliferation and osteoblastic differentiation on BCP or BG granules. Bone-associated morphological changes and gene expression modifications were quantified using histological and molecular techniques. The *in vivo* study was based on the subcutaneous implantation in nude mice of the biomaterials, loaded or not with one of the two cell types. Eight weeks after, bone formation was assessed using histological and electron microscopy techniques.

**Results:** Both cell types—BMMSC and NE-MSC—display the typical stem cell surface markers—CD73+, CD90+, CD105+, nestin - and exhibit the MSC-associated osteogenic, chondrogenic and adipogenic multipotency. NE-MSCs produce less collagen and alkaline phosphatase than BMMSCs. At the transcript level, NE-MSCs express more abundantly three genes coding for bone sialoprotein, osteocalcin and osteopontin while BMMSCs produce extra copies of RunX2. BMMSCs and NE-MSCs adhere and survive on BCP and BG. *In vivo* experiments reveal that bone formation is only observed with BMMSCs transplanted on BCP biomaterial.

**Conclusion:** Although belonging to the same superfamily of mesenchymal stem cells, BMMSCs and NE-MSCs exhibit striking differences, *in vitro* and *in vivo*. For future clinical applications, the association of BMMSCs with BCP biomaterial seems to be the most promising.

## Highlights

- Comparison of osteo-induction capacity of human adult bone marrow mesenchymal stem cells (BMMSCs) vs. child nasal ecto-mesenchymal stem cells (NE-MSCs) on BCP and BG biomaterials
- Both MSC subtypes adhere, proliferate, differentiate in osteoblasts and produce a mineralized extracellular matrix *in vitro*
- BMMSCs associated to BCP are more prone to induce bone formation *in vivo*
- Variations in osteoblastic gene expression may explain such a discrepancy

## Introduction

Biomaterial- and stem cell-associated bone repair represents a very promising alternative to autologous bone grafting. The most commonly investigated cells are mesenchymal stem cells (MSCs) (Hosseinpour et al., 2017; Luby et al., 2019; Hutchings et al., 2020; Donsante et al., 2021; Raghav et al., 2022). Among them, bone marrow-derived MSCs (BMMSCs) tend to be considered as the gold standard due to their capacity, in combination with biomaterial scaffolds, to repair bones *in vivo* (Arthur et Gronthos, 2020; Khorasani et al., 2021) However, other sources of MSCs have been poorly investigated (Gonzalez-Vilchis et al., 2022). Our laboratory identified and purified, from the *lamina propria* of the human olfactory mucosa, ecto-mesenchymal stem cells that originate from the neural crest and reside in the craniofacial area during adulthood (Féron et al., 2013; Veron et al., 2018). When compared to BMMSCs, nasal ecto-mesenchymal stem cells (NE-MSCs) display an inclination to differentiate into osteoblasts, rather than chondrocytes or adipocytes, and produce mineralized bone tissue *in vivo* after being filled on calcium phosphate ceramic discs and implanted subcutaneously in nude mice (Delorme et al., 2010).

Many biomaterials have been developed to offer surgeons and patients safe alternatives to autologous bone graft, a limited resource that adds morbidity at the harvesting site. The most commonly used are composed of biphasic calcium phosphate (BCP), a mixture of hydroxyapatite (HA)/beta-tricalcium phosphate (βTCP), similar to the mineral bone composition, but they display an insufficient osteoinductive capacity to regenerate large bone defects. BMMSCs, inserted in porous calcium phosphate ceramics, induce bone formation when implanted under the skin of nude mice or in rat or pig femurs (Dahl et al., 2014; Bouler et al., 2017; Ebrahimi et al., 2017). This BMMSC + BCP combination has even demonstrated safe bone regeneration in clinical trials (Hosseinpour et al., 2017).

Most recently, a synthetic bioactive glass (BG) or bioglass (bioglass 45S5 or GlassBONE™) has also been used for bone regeneration in orthopaedic, traumatic, spinal and cranio-maxillo-facial surgery (Profeta et al., 2016; Cannio et al., 2021; Liang et al., 2021). Based on borate and borosilicate, BG enhances bone and underlying neocartilage formation when compared to silicate bioactive glass (Rizwan et al., 2017). Moreover, borate-based BG displays controllable degradation rates and can be doped with trace quantities of elements such as Cu, Zn, and Sr, which are known to be beneficial for healthy bone growth. In addition, it has been observed that BG promotes angiogenesis which is critical in bone tissue regeneration (Kargozar et al., 2018).

Bone repair induced by stem cells and biomaterials may represent an alternative to autologous bone grafting. Numerous studies have demonstrated the effects of stem cells and biomaterials association on bone repair. However, studies using NE-MSCs and biomaterials are scarce, especially as a potential source of cells for repairing cleft palates. For that purpose, we designed a study comparing the osteogenic capacity of human mesenchymal stem cells (hMSC), derived from either adult bone marrow or newborn nasal cavity, associated with BCP or BG biomaterials, *in vitro* and after *subcutis* implantation in nude mice.

## Materials and Methods

### Donors

Bone marrow aspirates were obtained from the iliac crest by standard puncture and aspiration of healthy human male donors (21 and 26 years old), after having provided informed consent, according to the Declaration of Helsinki. The project approved by the Ethical Committee of ULM University. GMP grade BMMSCs were expanded according to previously published protocols (Brennan et al., 2014). In brief, BMMSCs were isolated from heparinized bone marrow aspirates by seeding 50,000 white blood cells/cm^2^ on two-chamber CellStacks (Corning/VWR, Ulm, Germany) in α-MEM culture medium (Lonza, Basel, Switzerland) supplemented with 5% GMP-grade human platelet lysate (PL) (IKT, Ulm, Germany) in order to avoid animal products. Cells were cultured for 10 or 14 days and the culture medium was changed twice per week. Cells were passaged at the density of 4,000 BM-MSCs/cm^2^ on two-chamber CellStacks in α-MEM supplemented with 8% PL for a further 5 or 7 days.

Male newborn NE-MSCs, purified from the nasal mucosa, were collected during a scheduled intervention for closing a cleft palate, under general anaesthesia. The procedure was approved by an ethical committee (CPP, RCB: 2015-A00984-45). Two infants were included in this pilot study. Biopsies were sliced in small pieces and incubated in 1.5 ml of collagenase (NB5, 1 U/ml, Nordmark Biochemicals) for 60 min at 37°C, before being mechanically dissociated. After digestion, 7 ml of serum-containing culture medium were added. After centrifugation, the cell pellet was resuspended in DMEM/HAM culture medium supplemented with antibiotics (penicillin/gentamicin and fungizone, Gibco™), and fetal calf serum (FCS) (Thermo Fisher Scientific, Waltham, Massachusetts, United States) on 25 cm^2^ plates. After 10 days, the cells were replated at the density of 4,000 cells/cm^2^ on 175 cm^2^ plates. At confluency, cells were detached with trypsin/EDTA 0.05% (Thermo Fisher) and collected NE-MSCs were stored in liquid nitrogen. Banking of stem cells for scientific research was performed anonymously, according to the rules of the Ministry of Higher Education and Research (number N DC-2011-1331).

### hMSC Characterization by Flow Cytometry

hMSC characterization by flow cytometry was performed on cells before seeding on biomaterials to demonstrate their characteristics as defined by the ISCT criteria (Dominici et al., 2006). hMSC were stained with CD90-FITC, CD73-PE, CD105-PC7, CD45-APC-A750 antibodies (Beckman Coulter, Brea, California, United States), or corresponding isotype controls in matched concentration, 20 min at room temperature (RT) and in the dark. Cells were washed in PBS without Ca^2+^/Mg^2+^ (Thermo Fisher), fixed, and permeabilized (IntraPrep Permeabilization Kit, Beckman Coulter). Intracellular staining was performed by an indirect immunofluorescence method with an adapted dilution of Nestin antibody (Merck Millipore, Burlington, Massachusetts, United States), or its corresponding isotype control in matched concentration, and with an Alexa Fluor 647 goat anti-mouse IgG (H + L) secondary antibody (Thermo Fisher). After washing, cells were analyzed with a NAVIOS flow cytometer (Beckman Coulter) and data files were interpretated using Kaluza software (Beckman Coulter).

### Tri-Lineage Differentiation Capacity of Expanded MSCs

- *Osteogenic differentiation*: both BMMSCs and NE-MSCs were plated at the density of 5 × 10^3^ cells/cm^2^ in 24 well plates in basal media. After 1 day, MSC differentiation towards an osteogenic lineage was induced with standard osteogenic supplements (10 mm β-glycerol-phosphate, 250 μm ascorbic acid, and 100 nm dexamethasone). At D7/14/21, cells were fixed with 4% paraformaldehyde (PFA) and mineralization was assessed by staining with a 40 mm alizarin red solution (pH 4.1–4.3).
- *Adipogenic differentiation*: MSCs were plated at the density of 2 × 10^4^ cells/cm^2^ in 24 well plates. Cells were cultured until reaching 80% confluency in basal media and then induced towards adipogenic lineage with the StemPro™ Adipogenesis Differentiation kit (Thermo Fisher). At D14/21, cells were fixed with 4% PFA and adipocytes stained with Oil Red O solution in 2-propanol diluted to 60%, using deionized water.
- *Chondrogenic differentiation*: collected MSCs were loaded into 15 ml tubes (5 × 10^5^ cells/tube) in fresh basal media and centrifuged at 1,500 rpm during 5 min. After 24 h, the basal medium was removed and StemPro™ Chondrogenesis Differentiation kit (Thermo Fisher) was added to the cell pellet. At D14/21, cell pellets were fixed in 4% PFA, and embedded in paraffin. Fixed pellets were cut with a microtome and stained with Alcian blue (Merck KGaA).

### Biomaterials

Two biomaterial scaffolds were compared: macro/microporous BCP granules 0.5–1 mm in size (MBCP+^®^, Biomatlante, Vigneux de Betagne, France), and BG granules 0.5–1 mm in size (GlassBONE™, Noraker, Villeurbanne, France). BCP is a ceramic composed of HA/βTCP in a ratio of 20/80 by weight. BG 45S5 composed of 45 wt.% SiO_2_, 24.5 wt.% Na^2^O and 24.5 wt.% CaO (Rizwan et al., 2017).

### Bioactive Glass Pre-Incubation

BG granules were incubated in 1 ml of phosphate buffered saline (PBS) for 5 h, leading to a highly basic pH 10. In order to get a neutral pH, the BG granules (90 mg) were incubated overnight in 1 ml of a calcium phosphate supersaturated solution (CPS), including 4 mm CaCl_2_.2H_2_O and 2 mm of Na_2_HPO_4_.2H_2_O in 0.9% NaCl buffered at pH 7.4 with TRIS/HCl. After an overnight soaking in CPS, the pH of the supernatant remained at 9. Based on these results, it was decided to seed the cells directly on the biomaterials without pre-incubation.

### Culture of MSCs With Biomaterials

Culture of human BMMSCs and NE-MSCs with biomaterials performed in Corning^®^ Costar^®^ ultra-low attachment 24 well plates (Merck KGaA, Darmstadt, Germany) with α-MEM (Thermo Fisher), supplemented with 8% human PL and 1% penicillin/streptomycin for 2–3 weeks (50 mg BCP or 90 mg BG per well). Cell seeding was done in static condition with 200,000 cells suspended in 250 μl of culture medium and seeded on 50 mg of BCP or 90 mg of GB per well in 24-well ULA plate (Corning^®^). The quantity of biomaterials was adapted to cover the entire surface of the well based on their respective density. The volume of cell suspension was adjusted for homogeneously seeding the biomaterial granules.

### Adhesion of MSCs on Biomaterials

200,000 cells were seeded on biomaterials in 250 μl of culture medium in 24 well ULA plate. After 1 h, 250 μl of medium was added. After 24 h, 500 μl of medium were added. The medium was changed 1 day after, and then every 3 days. Adhesion was assessed using TM3000 scanning electron microscope (Hitachi, Krefeld, Germany), operating at an acceleration voltage of 5 kV, and imaged at a magnification of 50×-500×, after fixation and dehydration of the cells in ethanol.

### Viability of MSCs

20,000 cells per well (1.05 × 10^4^ cells/cm^2^) were seeded in 24 well plate in basal media. Cells were seeded in 0.5 ml of media and the media was changed after 24 h. Then, media were changed every 3 days. Cell viability on biomaterials was evaluated at D2/7/14/21 by staining live cells with the fluorescent green stain calcein (1.25 μl/ml) and dead cells with fluorescent red ethidium homodimer-1 (1 μl/ml), according to Invitrogen™ L-3224 kit recommendations (Thermo Fisher).

### Proliferation of MSCs

Cell proliferation was assessed for 21 days using alamarBlue^®^ assay (Thermo Fisher). BMMSCs and NE-MSCs were seeded on 2D plastic (respectively at 500 and 5,000 cell/cm^2^), and on BCP and BP biomaterials. DNA assay has been done to calculate the relative amount of ALP normalised per cell. See section “ALP quantification”: At D1 and D7, MSCs were lysed using 0.1% Triton x-100, 5 mm Tris-HCL pH8 solution. After three freeze/thaw cycles, the amount of double stranded DNA was measured in the supernatants, using a fluorescent Quant-iT Picogreen dsDNA Assay kit (Thermo Fisher).

### Collagen Production

Collagen was visualized at D7/14/21 with Sirius red staining after culturing both cell types in proliferation or osteogenic culture media as previously described. Cells were fixed with 4% PFA in PBS for 20 min at RT, washed two times in PBS and stained with 1 ml of 1 mg/ml of Direct red solution (Merck KGaA) in saturated aqueous solution of picric acid (Merck KGaA), for 1 h at RT.

### Extracellular Alkaline Phosphate

ALP was qualitatively evaluated at D7/14/21 with Fast-Violet B Salt and Naphthol-AS-MX ALP staining (Merck KGaA). Cells were fixed with a solution, containing two volumes of citric acid-sodium citrate (1.5 mol/l) and three volumes of acetone for 30 s at RT. Cells were rinsed with deionized water and incubated, for 30 min in the dark, with a 50 ml staining solution. Including 48 ml of distilled water, 12 mg of Fast-Violet B Salt (F1631, Merck KGaA), and 2 ml of Naphthol-AS-MX ALP solution (855, Merck KGaA).

### ALP Quantification

At D1 and D7, MSCs were lysed using 0.1% Triton x-100, 5 mm Tris-HCL pH8 solution. After three freeze/thaw cycles, the amount of double stranded DNA was measured in the supernatants, using a fluorescent Quant-iT Picogreen dsDNA Assay kit (Thermo Fisher). The amount of ALP was measured using SigmaFast™ p-Nitrophenyl phosphate (pNPP) tablets (Merck KGaA). A standard curve was established with serial dilutions of pNPP and a known quantity of ALP from bovine intestinal mucosa (Merck KGaA). A known amount of pNPP was added to each sample, prior to incubation, during 30 min at 37°C. The amount of product (p-nitrophenol) was determined by reading the absorbance at 405 nm, on a microplate reader, and the amount of ALP was quantified using the following equation:

ALP(U/mL)=A/V/T

were *A*, pNPP in μmol; *V*, volume of sample in *mL*; *T*, time of incubation in minutes.

### Osteoblastic Differentiation by Real Time Quantitative PCR

Total RNA was extracted from cultivated cells with DirectZol™ RNA MiniPrep (Zymo Research, Irvine, California, United States) at D1 and D7. The RNA samples (1 μg each) was reverse transcribed with the maxima H minus First Strand cDNA Synthesis kit (Thermo Fisher) and oligo-dT primers in a final volume of 20 μl. The expression of each target gene was normalized with glyceraldehyde 3-phosphate dehydrogenase (GAPDH) and beta-2-microglobulin (B2M). The 2−^ΔΔCt^ method was used to calculate relative expression levels. The relative gene expression was normalized at D1 on plastic. The expression of the different bone-coding genes was assessed by RTqPCR.

### Primers

RUNX Family Transcription Factor 2 (*human RUNX2/F: gcc​tag​gcg​cat​ttc​aga*; *human RUNX2/R: gct​ctt​ctt​act​gag​agt​gga​agg*), Bone sialoprotein (*human BSP/F: caa​tct​gtg​cca​ctc​act​gc*; *human BSP /R: cag​tct​tca​ttt​tgg​tga​ttg​c*), Collagen Type I Alpha 1 Chain (*human COL1A1/F: ctg​gac​cta​aag​gtg​ctg​ct*; *human COL1A1/R: gct​cca​gcc​tct​cca​tct​tt*), Osteocalcin (*human OC/F: tga​gag​ccc​tca​cac​tcc​tc*; *human OC/R: ctg​gag​agg​agc​aga​act​gg*), Bone morphogenetic protein 2 (*human BMP2/F: gtt​cgg​cct​gaa​aca​gag​ac; human BMP2/R: cca​acc​tgg​tgt​cca​aaa​gt*), Bone morphogenetic protein 4 (*human BMP4/F: tca​aga​ttg​gct​gtc​aag​aat​gat​g*; *human BMP4 /R: cag​gta​tca​aac​tag​cat​ggc​tcg*), Alkaline phosphatase (*human ALP/F: aac​acc​acc​cag​ggg​aac*; *human ALP/R: ggt​cac​aat​gcc​cac​aga​tt*), and Osteogenic protein (*human OP/F: gag​ggc​ttg​gtt​gtc​agc*; *human OP/R: caa​ttc​tca​tgg​tag​tga​gtt​ttc​c*).

### *In Vivo* Experiments

The *in vivo* experiment was conducted according to the European regulation (Directive 2010/63/UE). The approval of the study was obtained from the local ethical committee on animal experiment (number6575). Eighteen 7 week-old female nude mice were used (NMRI nu/nu, Janvier Labs). Six groups were considered: BCP; BMMSC + BCP; NE-MSC + BCP; BG; BMMSC + BG; NE-MSC + BG on 50 mg of BCP or 90 mg of BG. No pre-treatment was performed with BG granules. 2 × 10^6^ cells were seeded on biomaterial granules and incubated overnight at 37°C with 5% CO_2,_ prior to implantation. Two identical subcutaneous implantation per mouse were performed. For statistical purpose, six implants per group were considered. Animals were operated under general anaesthesia with Isoflurane (Abbvie, North Chicago, Illinois, United States). A centimetric skin incision was performed on each side of mouse spine. Subcutaneous pockets were filled with granules, embedded with or without MSCs. Wounds were closed with non-resorbable suture 4.0 Filapeau (Péters Surgicals, Bobigny, France). Animals were controlled every day post-surgery, to monitor the healing of the skin and behavioural anomalies. Eight weeks post-implantation, animals were euthanized, and implants were fixed in 4% PFA.

### Histology

BCP and BG samples were dehydrated and embedded in poly-methyl-methacrylate resin (Merck KGaA). Sections were performed with Leica SP1600 microtome (Wetzlar, Germany), and bone formation was visualized by SEM. BCP samples were decalcified in Decalc (Microm Microtech, Brignais, France), dehydrated in ascending series of alcohol, and embedded in paraffin 4 μm-thin sections were cut with a microtome in the middle of the implant. Sections were stained by Masson trichrome in order to visualise newly formed bone tissue, granules, fibrosis, vascularization and cells. Toxicity and biodegradation of biomaterials were also determined.

### Statistics

When possible, numerical variables are reported by mean ± standard deviation. Qualitative variables are reported by frequency and percentage. Data processing and statistical analyses were performed with XLSTAT software (Microsoft, Redmond, Washington, United States). *p*-values less than 0.05 were considered significant. The results were expressed as mean ± standard deviation (SD).

## Results

### Newborn Nasal Stem Cells Display an NE-MSC Phenotype

**FIGURE 1:**
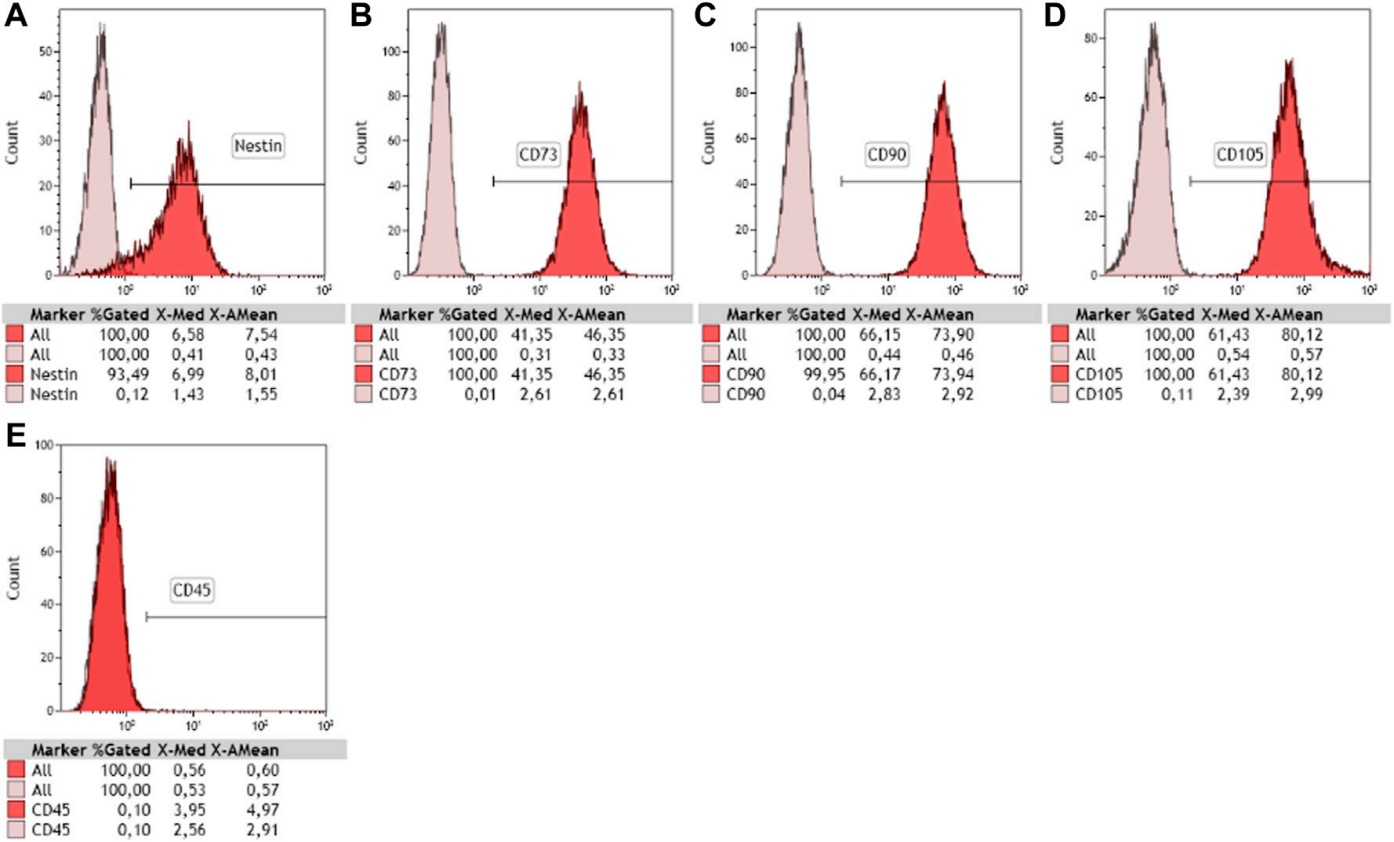
Characterization of newborn nasal stem cells. Phenotyping of cultivated cells was performed using flow cytometry and stem cell-specific markers. Purified nasal ecto-mesenchymal stem cells (NE-MSCs) were positive for nestin **(A)**, CD73 **(B)**, CD90 **(C)**, and CD105 **(D)**, recognized markers of mesenchymal stem cells, and negative for CD45 **(E)**, known marker for hematopoietic stem cells. 10,000 events counted.

Human newborn nasal cells being cultivated for the first time in the team, we first assessed their stemness. NE-MSCs express nestin (Figure 1A), a widely recognized marker for stem cells. In addition, they produce CD73, CD90 and CD105 (Figures 1B–D), as documented for adult nasal olfactory ecto-mesenchymal stem cells, and not CD45, a specific hematopoietic stem cell marker (Figure 1E).

### *In Vitro* Assessment of Stem Cell Multipotency

**FIGURE 2:**
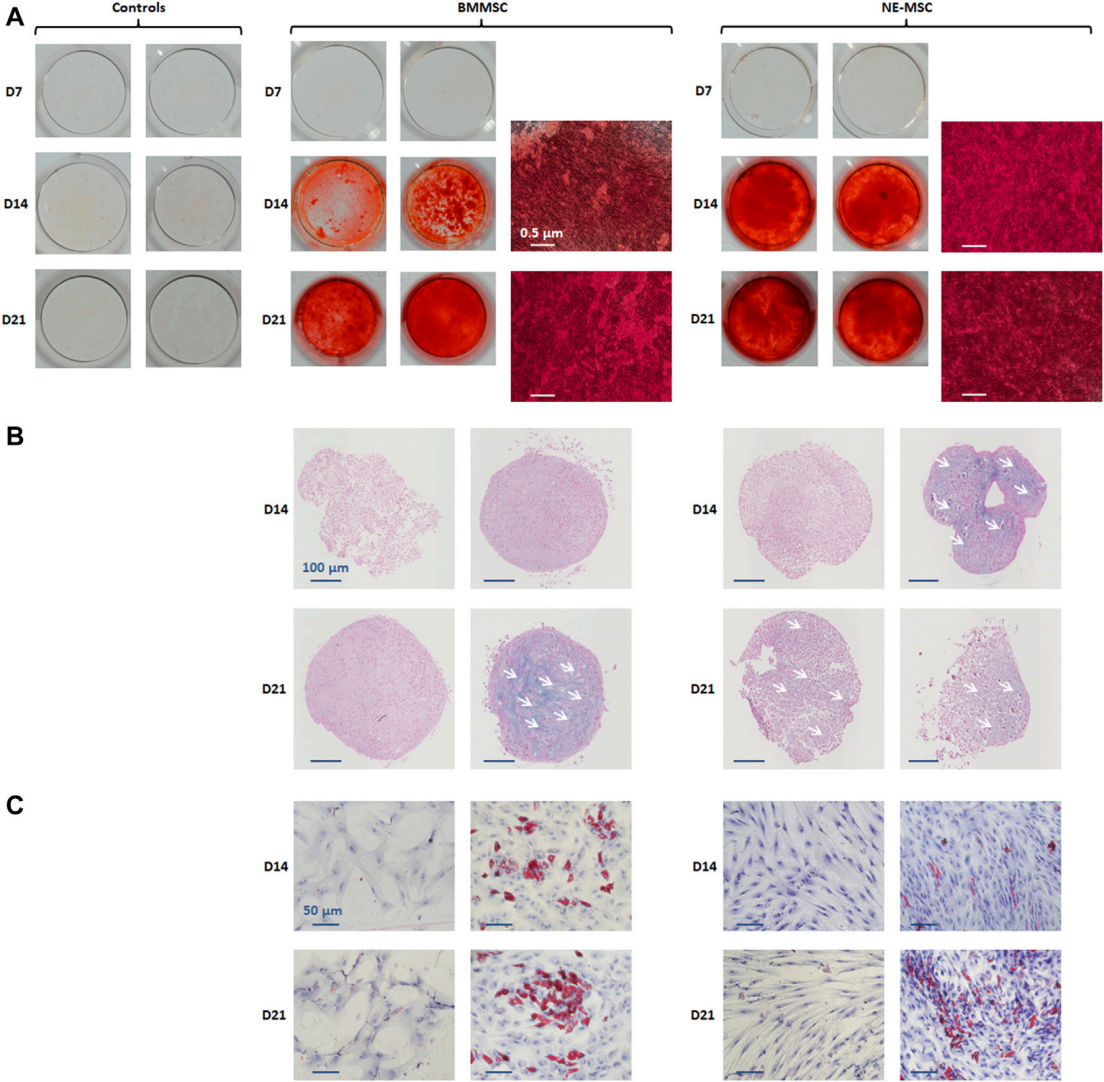
*In vitro* assessment of multipotency. BMMSCs and NE-MSCs were induced to differentiate into osteoblasts (alizarin red, **(A)**, chondroblasts (alcian blue, **(B)** and adipoblasts (red oil, **(C)**). Both BMMSCs and NE-MSCs displayed mineralization production at D14 (red cells). NE-MSCs differentiated in chondroblasts at D14 and BMMSCs at D21 (blue cells). BMMSCs and NE-MSCs also differentiated in adipoblasts at D14 (red cells).

We compared the capacity of both MSC types to differentiate *in vitro* into osteoblasts, chondroblasts and adipoblasts, using the usual stains: alizarin red (Figure 2A), alcian blue (Figure 2B), and red oil (Figure 2C). BMMSCs and NE-MSCs display an intense mineralization at D14 and exhibit a similar pattern of differentiation for chondroblasts and adipoblasts.

### BMMSCs and NE-MSCs Adhere and Survive on Biomaterials

**FIGURE 3:**
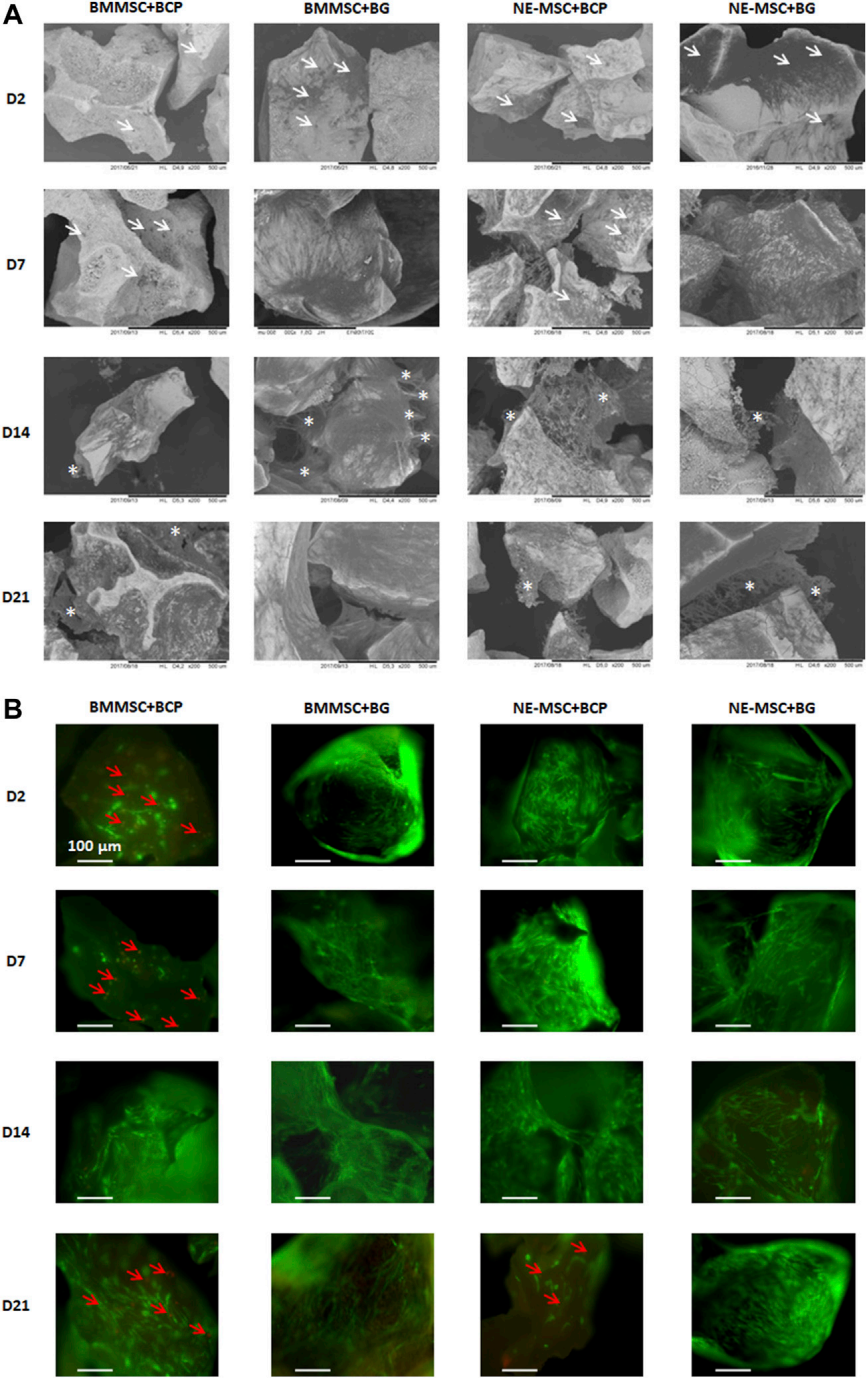
**(A)** Comparative adhesion of the two MSC types on biomaterials using SEM. Both BM-MSCs and NE-MSCs rapidly adhere to BCP and BG (white arrows) and synthesize an extracellular matrix at D14 (white asterisks). **(B)** Comparative viability of the two MSC types on biomaterials using fluorescent stains for living (green) and dead (red) cells (red arrows). BG, and not BCP, induces cell death, as early as D2 for BM-MSCs, and only at D21 for NE-MSCs.

BCP and BG had similar shape and size but different surface microstructures in SEM, BCP particles showed microporosity, whereas BG exhibited a smooth surface (Figure 3A). Both BMMSCs and NE-MSCs adhere to BCP and BG, more rapidly to BG than to BCP (white arrows). BMMSCs synthesized an extracellular matrix after D14, which seems to be more abundant on BG than on BCP, but mineralization was only observed on BCP (white asterisks). NE-MSCs produced also an abundant but fragile mineralized extracellular matrix on both biomaterials, less on BG than on BCP (white asterisks).

Using fluorescent stains for living (green) and dead (red) cells, BMMSCs and NE-MSCs viability was kinematically assessed. Dead cells (red arrows) are observed only on BCP biomaterial. Death of NE-MSCs was only observed at D21 while apoptosis/necrosis of BMMSCs starts as soon as D2, a possible indication that NE-MSCs are more resistant to a relatively adverse material.

### BMMSCs Display a Higher Proliferation on BG Biomaterial

**FIGURE 4:**
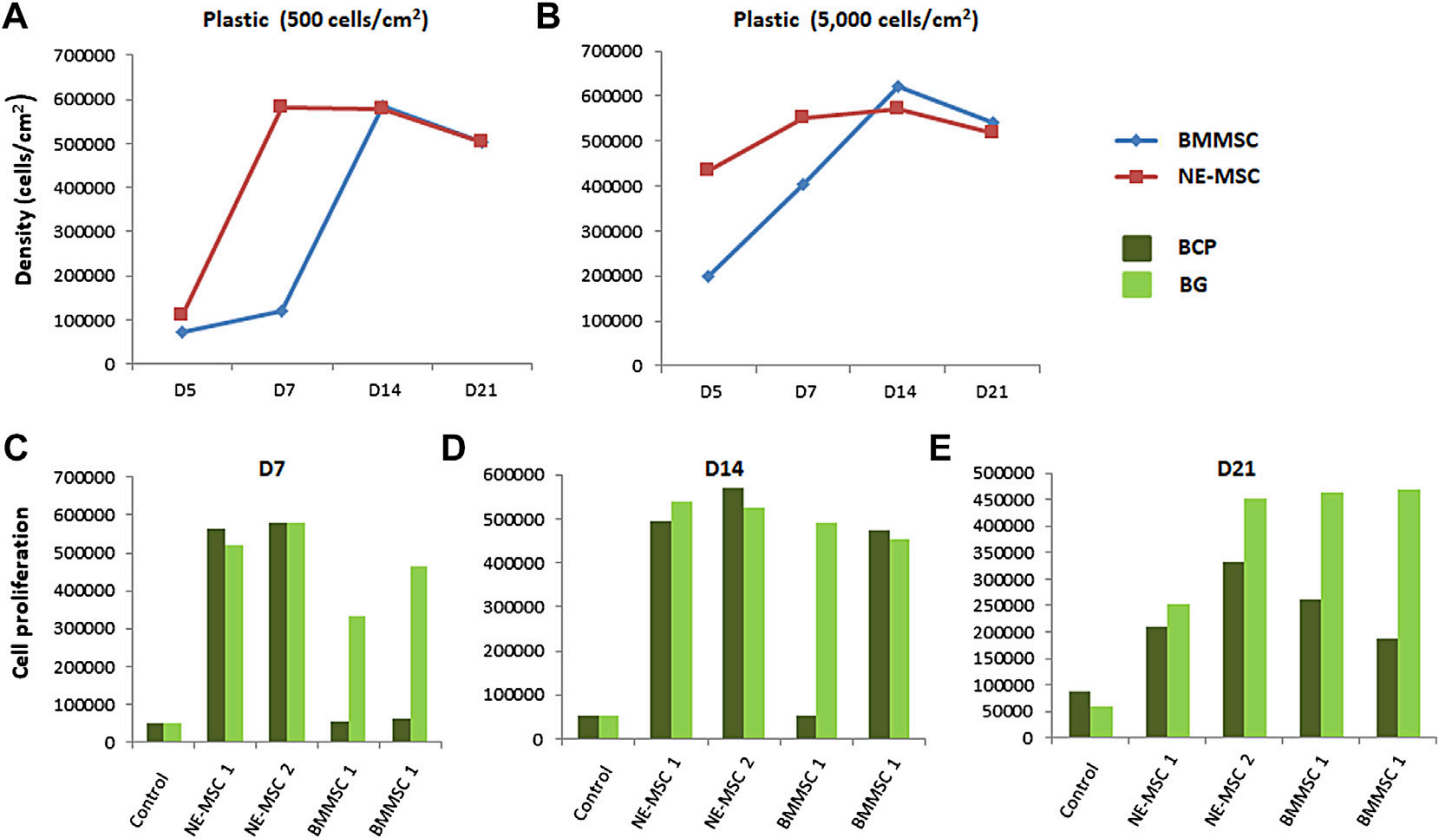
Influence of the biomaterial on cell proliferation. BMMSCs and NE-MSCs were seeded on 2D plastic at two different densities: 500 **(A)** and 5,000 cell/cm^2^ **(B)**, on BCP and BP biomaterials **(C–E)**, and cell metabolic activity monitored over a period of 3 weeks using alamarBlue^®^ assay. On plastic, BMMSCs and NE-MSCs display a high proliferation rate. On plastic and on both biomaterials, NE-MSCs proliferate more rapidly than BMMSCs. BG biomaterial favors BMMSC proliferation. Controls correspond to cell-free biomaterials (background noise of alamar blue).

On plastic, BMMSCs and NE-MSCs display a high proliferation rate, although stem cells from newborns outweigh adult stem cells (Delorme et al., 2010). It was then questionable to confirm this finding when using biomaterials. Figure 4 indicates that, on both biomaterials, NE-MSCs proliferate more rapidly than BMMSCs at the start of the experiment. However, at D14, the numbers are even and, subsequently, due to cell death occurring at this stage (see above), the density of both MSCs declines (Figures 4A,B). Figures 4C–E indicate that BG favors BMMSC proliferation, a result in line with an increased viability of BMMSC viability on BG, when compared to BCP (Figure 3B). No difference between the two materials was observed for NE-MSCs.

### Collagen Production Increases in BMMSCs and Decreases in NE-MSCs

**FIGURE 5:**
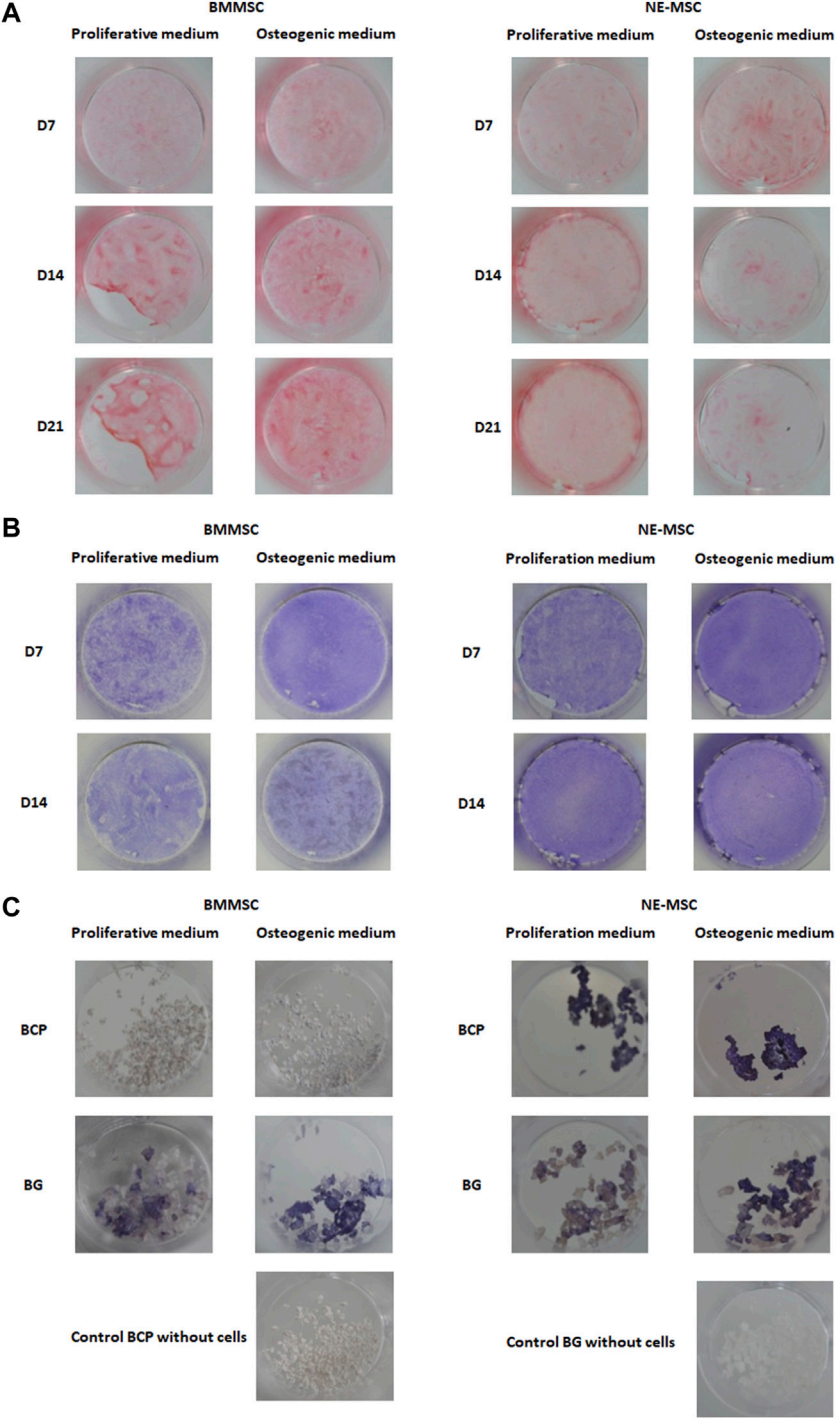
**(A)** Comparative production of collagen. In osteogenic conditions, BMMSCs increasingly produce collagen while the opposite is observed for NE-MSCs. Comparative production of ALP by both cell types, at D7. BMMSCs and NE-MSCs were cultivated either on plastic **(B)** or biomaterials **(C)**. BMMSCs express ALP only when cultivated on BG biomaterial while NE-MSCs produce ALP on both biomaterials. For NE-MSCs, ALP production is higher in proliferative medium.

BMMSCs produce more collagen, a chondrocytic and osteogenic specific marker (Sirius red staining), than NE-MSCs. Noticeably, collagen expression timely increases in BMMSCs and declines in NE-MSCs (Figure 5A).

### BMMSCs Produce ALP Only on BG Biomaterial

**FIGURE 6:**
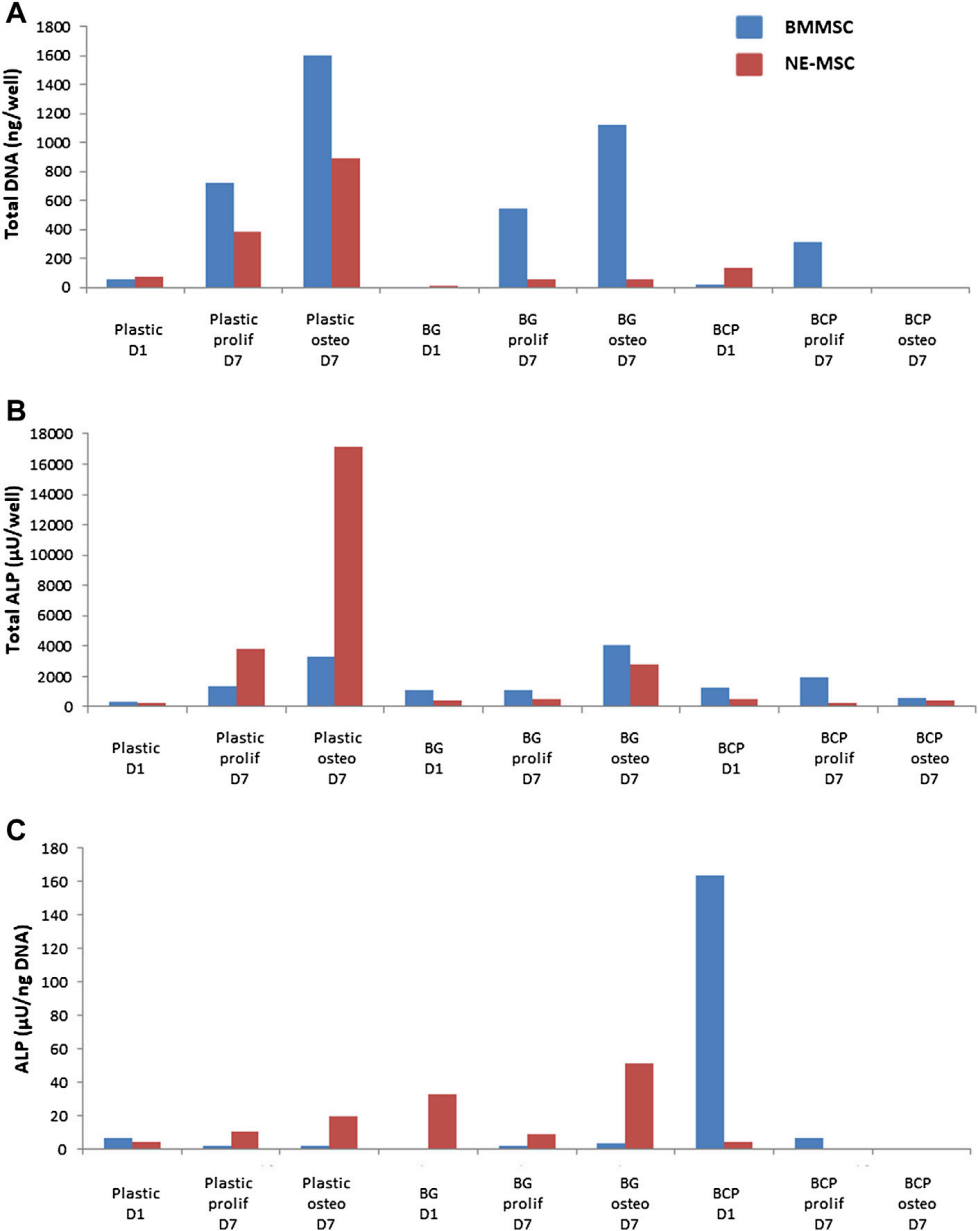
Quantification of alkaline phosphatase (ALP). For the purpose of comparison, the total amount of DNA, at D1 and D7, was quantified for both cell types, BMMSCs and NE-MSCs, in proliferative and osteogenic conditions **(A)**. The total amount of *ALP* in each condition was assessed **(B)** and then compared to the amount of DNA **(C)**. NE-MSCs express an important amount of *ALP* when grown on BG, in osteogenic conditions. For BMMSCs, *ALP* expression is marginal except at D1 on BCP biomaterial.

BMMSCs and NE-MSCs produced ALP on plastic (Figure 5B). At D7, BMMSCs express ALP only when cultivated on BG, while NE-MSCs produce ALP on both biomaterials with a higher production in proliferative medium (Figure 5C). To move further and quantify ALP production in various conditions, we assessed its expression in accordance with cell abundance (Figures 6A,B). NE-MSCs express an important amount of ALP when grown on BG in osteogenic conditions, and conversely is negligible on BCP, except at D1 (Figure 6C).

### A Differentiated Expression of Osteoblast-Associated Genes

**FIGURE 7:**
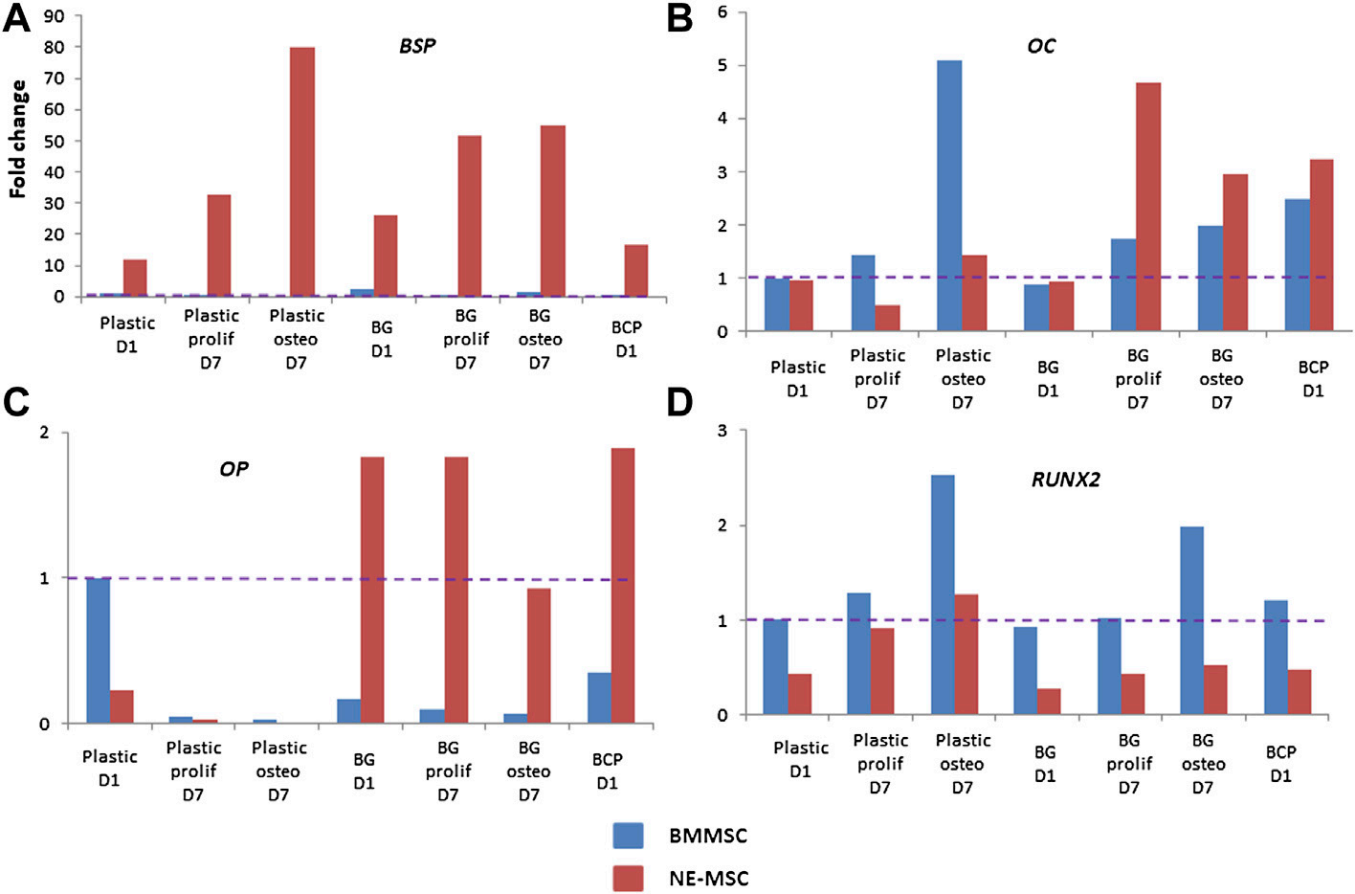
Comparative expression of ossification-related genes. Using RTqPCR, the expression of *RUNX2*, *BSP*, *COL1A1*, *OC*, *BMP2*, *BMP4*, *ALP*, and *OP* was measured for each cell type on plastic, BG and BCP biomaterials. On BG biomaterial, NE-MSCs overexpress the genes coding for BSP, OC and OP **(A–C)** and BMMSCs the gene coding for RUNX2 **(D)**.

**FIGURE 8:**
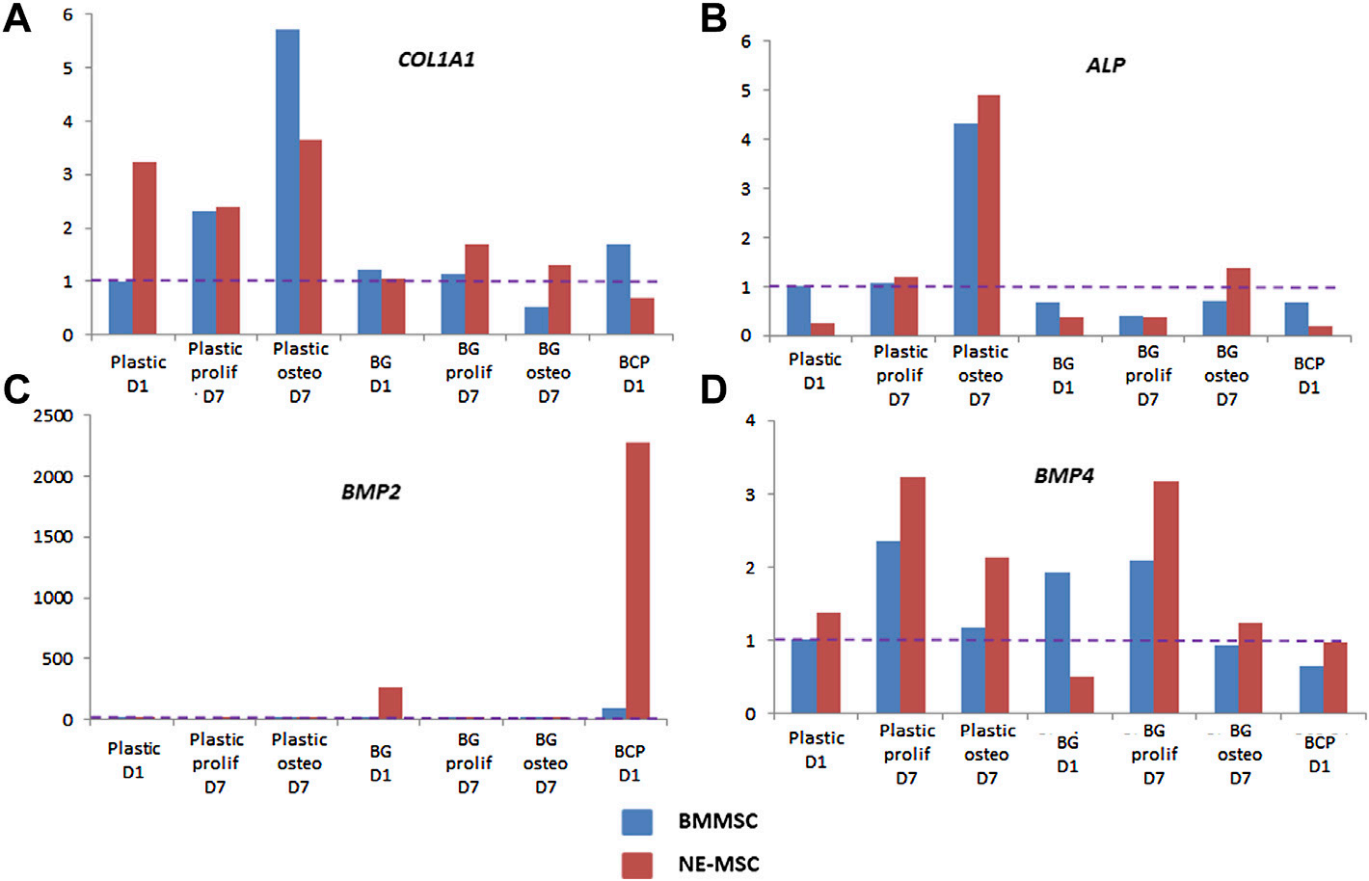
Expression of other ossification-related genes-*COL1A1* **(A)**, *ALP* **(B)**, *BMP2* **(C)**, and *BMP4* **(D)**-was measured by RTqPCR for each cell type on plastic, BG and BCP biomaterials. No data at D7 on BCP was obtained because the cells did not proliferate and the amount of RNA was too small.

Discrepancies between both MSCs further analyzed by measuring the expression of eight bone-associated genes at D1 and D7 on both biomaterials (Figure 7). Normalization of gene expression was performed using the amount expressed by BMMSCs at D1 on BCP, as basal level. When cultivated on BG, NE-MSCs overexpress the genes coding for BSP, OC and OP (Figures 7A–C). Conversely, on the same biomaterial, BMMSCs produce a higher number of transcripts coding for RUNX2 (Figure 7D). No significant difference between the two groups was noted for other genes (Figures 8A,C,D). ALP gene expression exhibited quite similar variations to ALP in culture supernatant, except for BCP at D1 (Figure 8B). No data are provided for BCP at D7 because a very small amount of RNA was extracted. This may be due to the failure of cell adhesion and proliferation on BCP, which can also explain the absence of correlation between ALP quantification and ALP gene expression at D1 on BCP. Indeed, reproducibility of experiment was affected by the absence of pre-incubation of BCP in culture medium prior to cell seeding.

### *In Vivo* Experiments

**FIGURE 9:**
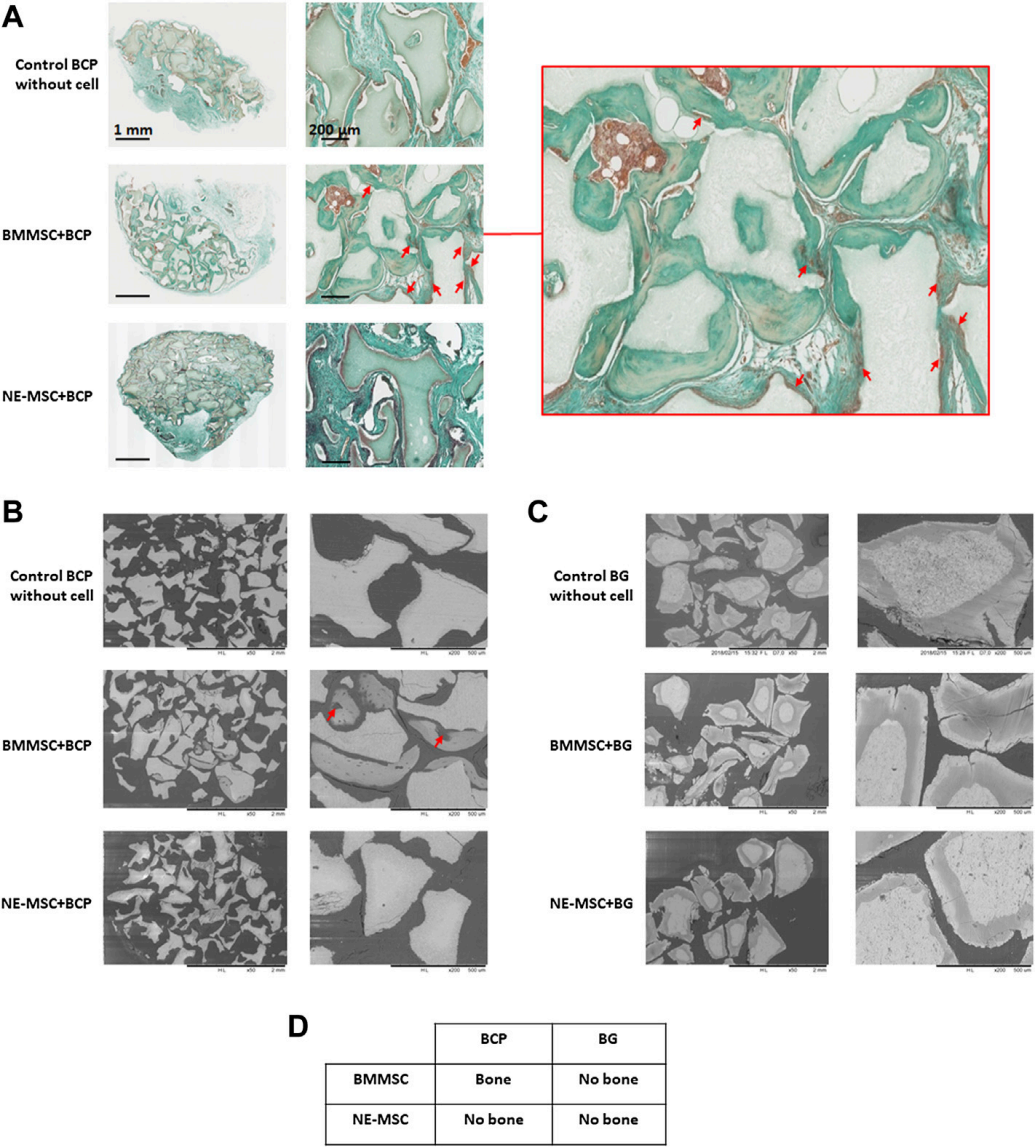
*In vivo* bone formation, 8 weeks after subcutaneous implantation in nude mice. **(A)** BMMSCs give rise to osteoblasts, stained with Masson trichrome (red arrows), but not NE-MSCs and cell-free BCP biomaterial. **(B)** The same finding is observed when the implants are analyzed with an electronic microscope (red arrows). **(C)** No bone formation is observed when cells were loaded in BG biomaterial. **(D)** Table summarizing the findings.

Eight weeks after subcutaneous implantation in nude mice, none of the cell-free BG was found to harbor bone tissue, the BG granules were only surrounded by vascularized fibrous tissue (Figure 9A). No bone formation was either observed on the same biomaterial loaded with BMMSCs or NE-MSCs (Figure 9C). BMMSCs gave rise to bone tissue when grafted on BCP (Figures 9A,B). Conversely, NE-MSCs failed to produce osteoblasts (Figures 9A,B). Figure 9D resumes *in vivo* findings. No sign of systemic or local toxicity, no infection, and no behavioral change were observed with both biomaterials, with or without MSCs.

## Discussion

In this study, we compared the *in vivo* osteoinduction potential of hMSCs derived from either bone marrow or child nose, associated with BCP or BG, two synthetic bone fillers currently used for their favorable bioactivity and osteoconductive properties (Ben Azouna et al., 2012; Leotot et al., 2013). Alone these biomaterials have insufficient osteoinductive properties to regenerate large bone defects. Combinations of hMSCs and BCP have been shown to induce *de novo* bone in ectopic sites and thus, have been widely studied *in vivo* as alternatives to autologous bone grafts (Leotot et al., 2013; Brennan et al., 2014; Gjerde et al., 2018). On the opposite, combinations of MSCs and BG have been mainly studied *in vitro*, but poorly *in vivo* (Liang et al., 2021).

Although belonging to the same superfamily, BMMSCs and NE-MSCs exhibit striking differences, *in vitro* and *in vivo*. For future clinical applications, the association of BMMSCs with BCP biomaterial seems to be the most promising. Indeed, our *in vivo* experiments reveal that osteoinduction is only observed when BMMSCs transplanted on BCP. Recently, Rodrigues and colleagues reported that allogenic adipose-derived MSCs associated with BG 1) is biocompatible in the long term (3 months), 2) maintain their osteoinduction potential, and 3) is safe after subcutaneous implantation in immunocompetent *balb-c* mice. Indeed, a low spreading during cell adhesion was detected associated with an increase of HA depositions around the cells that look like differentiated osteoblasts (Rodrigues et al., 2019). Any apparent local or systemic toxicity for organs or strong immunogenic reactions were noted, except a vascularized dense capsule around the graft (Rodrigues et al., 2019).

Certain *in vivo* conditions could significatively impact the engraftment and the bone formation. In our study, we chose to use nude mice to limit the immune response against the MSC allograft. The MSC allograft in Balb-c mice is possible, but the capsule formation surrounding the graft, probably due to the immune response in immunocompetent mice, could be a complication for implants (Velnar et al., 2016; Chung et al., 2017). The pH and the calcium concentration can also affect the cells. Here, we worked with 4 mm of calcium in culture medium. Maeno et al. (2005) showed that high concentration of calcium above 10 mm are cytotoxic to osteoblasts, but those below 8 mm promote cell proliferation. We also used medium supplemented with 5% GMP-grade human PL for MSC culture. It is now well established that animal derived products such as FCS could significantly interact with phenotypical and functional characteristics of BMMSCs (Ben Azouna et al., 2012; Leotot et al., 2013). Here, MSCs were grown with biomaterials 24 h before graft, and we implanted 2 × 10^6^ MSCs per site. In comparison, Rodrigues et al., (2019) used 2 × 10^4^ MSCs grown during 48 h on BG before implantation.

Several differences are also observed at the transcriptomic level: NE-MSCs express more abundantly three genes coding for bone sialoprotein, osteocalcin and osteopontin while BMMSCs produced extra copies of *RUNX2*. This can probably explain the difference of mineralization of the extracellular matrix between BM- and NE-MSCs. It would be interesting to upregulate these genes in NE-MSCs or to down regulate these genes in BMMSCs to observe what will happen in terms of composition and mineralization of the 3D constructs. Hence, by identifying the modulating expression of genes implied in mineralization, it would be possible to optimize human MSC culture conditions or biomaterials with specific coating modulating factors. In these conditions, it will be interesting to perform again the same type of study. But these patterns of gene expression were not sufficient to explain the difference of osteoinduction *in vivo*. It would also be interesting to determine the gene expression pattern of adipose-MSCs and to compare with that obtained with BM- and NE-MSCs. This would also have repercussions on the comprehension of pathological mechanisms as well as in osteoporosis or other degenerative bone disorders (Gambacurta et al., 2019; Jo et al., 2019) as well as bone tumors (Valenti et al., 2018).

Intriguingly, newborn nasal mesenchymal stem cells failed to differentiate into osteoblasts while adult nasal olfactory ecto-mesenchymal stem cells (OE-MSC) proved to be efficient for bone repair. They even outweigh bone marrow mesenchymal stem cells, when grafted in biphasic calcium phosphate ceramic discs (Delorme et al., 2009). In both studies, the elected biomaterial being identical, the reason for the discrepancy is likely associated to the main characteristics - age of the donor and location in the nose - of each stem cell subtype. Since newborn cells are usually more plastic than adult cells and display a higher differentiation potential (Chen et al., 2017), it was hypothesized that the former would perform at least as well as the latter. Our results indicate the opposite. Therefore, the location within the nasal cavity is probably the explanatory key. Nasal olfactory stem cells are now well characterized, and their differentiation potential exhaustively assessed, especially for brain repair (Duan & Lu, 2015). In contrast, the stem cells collected from the anterior part of the newborn nose are newcomers in the field of ecto-mesenchymal stem cell subfamily and need to be fully depicted.

The discordant findings between *in vitro* and *in vivo* experiments with NE-MSCs are also enigmatic. The biomaterial could hardly be blamed since adult OE-MSCs differentiated into osteoblasts on BCP ceramic discs (Delorme et al., 2009). Obviously, a further characterization of NE-MSCs is required to find a convincing explanation. It should however be pointed out that the *in vivo* study is not as exhaustive as the *in vitro* study when bone-specific proteins and minerals are considered. In addition, as highlighted in a review article describing the osteogenic differentiation of human MSCs, the modifications observed *in vitro* are not always translated when the cells are grafted in an animal (Mollentze et al., 2021).

Initially, this project was designed in view to treat cleft palate. These malformations affect the face: there is a communication between the nose and the mouth interrupting the lip till the bone. Management requires three surgical procedures in the first 5 years of life (Worley et al., 2018; Stein et al., 2019): the closing of the lip in the first days, then the palate, up to 1 year, and finally the bone gap, up to 4 years, by means of a bone graft. We think that NE-MSCs could promote bone regeneration in view to avoid the third operation. Unfortunately, NE-MSCs and BG are not the best candidates for osteoinduction. It seems preferable to use BMMSCs associated with BCP for this indication.

## Conclusion

Bone repair induced by stem cells and biomaterials may represent an alternative to autologous bone grafting. Here, we compared the efficiency of two biomaterials—biphasic calcium phosphate (BCP) and bioactive glass (BG)—when loaded with either adult bone marrow mesenchymal stem cells (BMMSCs) or newborn nasal ecto-mesenchymal stem cells (NE-MSCs), the latter being collected for further repair of lip cleft-associated bone loss.

*In vitro*, BMMSCs and NE-MSCs adhere and survive both on BCP and BG, BMMSCs display a higher proliferation on BG biomaterial. NE-MSCs produce a fragile mineralized extracellular matrix, with less collagen and alkaline phosphatase than BMMSCs. Variation in osteoblastic gene expression may explain such a discrepancy. *In vivo* experiments reveal that bone formation is only observed with BMMSCs transplanted on BCP biomaterial. The association of NE-MSCs with both biomaterials was not efficient and, pending further studies, is not yet recommended.

## Data Availability

The original contributions presented in the study are included in the article/supplementary material, further inquiries can be directed to the corresponding author.

## Abbreviations

MSC, mesenchymal stromal/stem cell; hMSC, human mesenchymal stem cell; BMMSC, bone marrow derived MSC; OE-MSC, olfactory ecto-mesenchymal stem cell; NE-MSC, nasal ecto-mesenchymal stem cell; GMP, good manufacturing practice; BCP, biphasic calcium phosphate; HA, hydroxyapatite; βTCP, beta-tricalcium phosphate; BG, bioactive glass; PBS, phosphate buffered saline; CPS, calcium phosphate supersaturated solution; RT, room temperature; PFA, paraformaldehyde; pNPP, p-Nitrophenyl phosphate; RTqPCR, real time quantitative PCR; GAPDH, glyceraldehyde 3-phosphate dehydrogenase; B2M, beta-2-microglobulin; RUNX2, RUNX family transcription factor 2; BSP, bone salioprotein; COLA1, collagen A1; OC, osteocalcin; BMP2, bone morphogenic protein 2; BMP4, bone morphogenic protein 4; ALP, alkaline phosphatase; OP, osteopontin; SEM, scanning electron microscopy; SD, standard deviation.
